# Improved Yield of High Molecular Weight DNA Coincides with Increased Microbial Diversity Access from Iron Oxide Cemented Sub-Surface Clay Environments

**DOI:** 10.1371/journal.pone.0102826

**Published:** 2014-07-17

**Authors:** Richard A. Hurt, Michael S. Robeson, Migun Shakya, James G. Moberly, Tatiana A. Vishnivetskaya, Baohua Gu, Dwayne A. Elias

**Affiliations:** 1 Biosciences Division, Oak Ridge National Laboratory, Oak Ridge, Tennessee, United States of America; 2 Department of Microbiology, University of Tennessee, Knoxville, Tennessee, United States of America; 3 Environmental Sciences Division, Oak Ridge National Laboratory, Oak Ridge, Tennessee, United States of America; Leibniz-Institute DSMZ, Germany

## Abstract

Despite over three decades of progress, extraction of high molecular weight (HMW) DNA from high clay soils or iron oxide cemented clay has remained challenging. HMW DNA is desirable for next generation sequencing as it yields the most comprehensive coverage. Several DNA extraction procedures were compared from samples that exhibit strong nucleic acid adsorption. pH manipulation or use of alternative ion solutions offered no improvement in nucleic acid recovery. Lysis by liquid N_2_ grinding in concentrated guanidine followed by concentrated sodium phosphate extraction supported HMW DNA recovery from clays high in iron oxides. DNA recovered using 1 M sodium phosphate buffer (PB) as a competitive desorptive wash was 15.22±2.33 µg DNA/g clay, with most DNA consisting of >20 Kb fragments, compared to 2.46±0.25 µg DNA/g clay with the Powerlyzer system (MoBio). Increasing PB concentration in the lysis reagent coincided with increasing DNA fragment length during initial extraction. Rarefaction plots of 16S rRNA (V1–V3 region) pyrosequencing from A-horizon and clay soils showed an ∼80% and ∼400% larger accessed diversity compared to the Powerlyzer soil DNA system, respectively. The observed diversity from the *Firmicutes* showed the strongest increase with >3-fold more operational taxonomic units (OTU) recovered.

## Introduction

The inability to extract nucleic acids (NA) from all populations within a microbial community in sufficient quantity and quality has limited the efficacy of phylogenetic studies in many environments. Nucleic acid extraction is a well-established source of bias concerning observed microbial communities, most often caused by variation in cell envelope characteristics; eg. Gram positive versus Gram negative bacteria [1], [2], [3]. When comparing extraction procedures, a higher DNA yield can indicate low sampling bias and thus a more complete assessment of the microbial community [4], [5]. However, NA preparation methods differ in NA loss to adsorption, making it difficult to judge microbial community coverage on the basis of NA yield. Because NA adsorption to the environmental matrix makes it impossible to judge extraction bias on the basis of yield, a comparison of procedures should be performed on the basis of the accessed microbial diversity.

For iron rich clays, DNA and RNA yields can be minimal or absent, primarily due to NA adsorption. The polyanionic property of NA deriving from the 5′-phosphate linkages supports a large number of binding sites with iron or other multivalent cations bound to negatively charged clay particles. Therefore, it is reasonable to anticipate that longer NA polymers will have a larger number of ionic interactions with substrates carrying multiple positive charges, and thereby have increased binding strength with iron oxides [6], [7]. While this stronger binding of long DNA polymers can bias recovery towards smaller DNA fragments, it is not likely to influence what microbial groups are identified, because DNA adsorption is non-selective with respect to polynucleotide content [8].

Carboxyl and hydroxyl groups of humic and fulvic acids form stable complexes with metal cations, with a binding strength order of Fe^3+^>Al^3+^>Pb^2+^>Ca^2+^>Mn^2+^>Mg^2+^ [9], while phosphate and sulfate reduce adsorption of natural organic matter on oxide minerals [9], [10]. A number of these metals, particularly Al^3+^, are prevalent in clay materials and can reduce microbial activity [11]. Further, different clay mineral structures have different adsorptive properties [12] including adsorption of macromolecules through multisite binding, hydrogen bonding and hydrophobic interactions [13] which offer a potential explanation for the observed differential DNA and RNA adsorption to environmental matrices.

A more comprehensive extraction method for attaining intact NA from clays would support exploration of environments currently considered recalcitrant to molecular analysis. Prior work targeting such a universal extraction procedure used 100 mM phosphate in the extraction buffer to limit adsorption or promote desorption [14], [15], [16]. Tanaka *et al.* showed that 1 M sodium phosphate supported efficient recovery of DNA from aminosilane-modified magnetic nanoparticles [17], and Direito *et al.* used 1 M sodium phosphate and heat to recover DNA from Mars analogue substrates in a bead milling process [18].

High molecular weight (HMW) DNA is required for current high throughput microbial community genome sequencing procedures for the same reasons that it is essential for the preparation of cloned metagenome libraries, and maximizing DNA fragment size by direct extraction is the focus of the work described here [19], [20]. DNA samples extracted directly from subsurface sediments taken from the Oak Ridge National Laboratory Field Site at East Fork Poplar Creek (EFPC) have presented a significant challenge, with all applied procedures yielding fragmented NA in low yield. A common problem for EFPC sediments is adsorption to iron oxide cemented clay particles. For liquid nitrogen grinding procedures, adsorption often results in a complete loss of released NA onto EFPC samples. The desorptive sediment wash described herein supported HMW DNA recovery from iron cemented clay environments while grinding sediment samples in the presence of concentrated guanidine and sodium phosphate showed a large improvement in accessed microbial diversity as opposed to the ORNL 2001 procedure [14]. These steps can easily be appended to any direct NA extraction procedure.

## Results

### Extraction process and modification

Stream sediment core samples from EFPC were not yielding DNA of sufficient quantity or quality for desired molecular analysis using either published methods or marketed kits. The stream sediment samples largely consisted of a mixture of gravel and iron cemented clay as indicated by a rust color after exposure to air. For method development, a deciduous forest A-horizon and its subsurface iron cemented clay were used. The clay sample had a uniform texture and a consistent moisture content of 24% at depths >15 cm below the surface. Initial NA extraction from iron cemented clay using a commercial bead milling process, or liquid nitrogen grinding with a urea based extraction buffer was not productive, and the similarity in color (red) to the refractory EFPC stream sediments suggested a common source of difficulty. The stream sediments contained coal fragments that caused a high percent of total carbon; however, the control site (Hinds Creek) consistently yielded a large quantity of co-extracted humic acid as well (Table S1). The A-horizon sample had the largest quantity of co-extracted organic matter however; coal fragments were not present resulting in a lower total carbon measurement (Table S1). Although the iron cemented clay had the lowest quantity of acid extractable iron (0.08 mg/g) it contained the highest total iron (38.66 mg/g) following acid digestion, demonstrating that essentially all available iron was in the form of crystalline iron oxides cemented with the clay. The surface (A-horizon) soil above the iron cemented clay was used as a positive control to insure that extraction systems were functioning to expectation. Like the subsurface clay sample, the A-horizon sample also contained a large amount of iron and consisted largely of iron cemented clay infiltrated with humus (Table S1).

**Figure 1:**
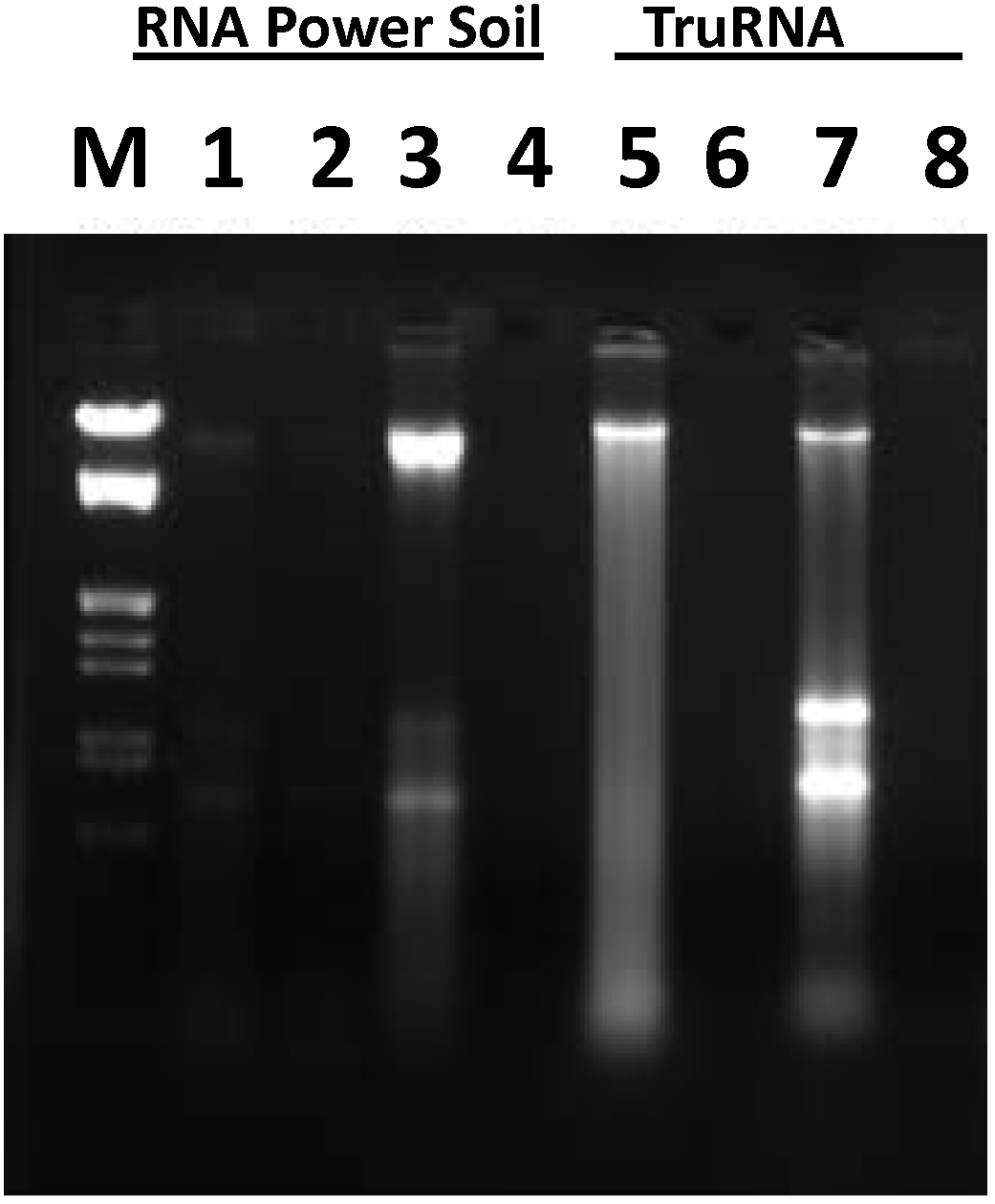
Reagent test and process development sample evaluation. Ethidium bromide stained 1.2% agarose TAE electrophoresis image (3 V/cm×90 min) comparing reagent systems used for stream sediment RNA or TNA recovery. Lane M; Marker III (Roche), lanes (1, 5) extraction product from NOAA sediment core, lanes (2, 6) extraction product from New Horizon sediment core, lanes (3, 7) extraction products from the A-horizon surface soil sample, lanes (4, 8) extraction products from the iron cemented clay sample.

Tests performed using a standard lysis solution (4 M guanidine isothiocyanate; 100 mM Tris pH 7.0, 20 mM EDTA, 0.6% sarkosyl, 10 µ l/ml 2-mercaptoethanol) yielded DNA from the deciduous forest A-horizon, but no detectable DNA from the iron oxide cemented clay (Figure 1). Triplicate extractions using the presently described ORNL 2012 reagents and procedures in combination with either the RNA PowerSoil™ (Figure 1, lanes 1–4) or *TruRNA*™ (Figure 1, lanes 5–8) kits supported the simultaneous recovery of DNA and RNA from the A-horizon soil (Figure 1, lanes 3, 7) and the RNA exhibited good 23S/16S rRNA stoichiometry indicating an absence of NA degradation. RNA PowerSoil reagents (MoBio) yielded some NA from one of the stream sediments (Figure 1, lane 1) but no detectable DNA from the second stream sediment (Figure 1, lane 2) or the iron cemented clay (Figure 1, lane 4).

The effect of pH on total NA (TNA) extraction from iron oxide cemented clay was tested over a range of 5.2 to 8.0. The entire range of standard Tris buffered lysis reagent, including unadjusted 200 mM Tris-HCl (pH 3.9) and unadjusted 200 mM Trizma base (pH 10.0), did not block NA adsorption to iron cemented clay. The effect of omitting sarkosyl from lysis reagent modified with 300 mM sodium phosphate buffer (PB) pH 7.2 resulted in a lower yield of DNA from iron cemented clay, while inclusion of 10% w/v BSA resulted in a larger quantity of extracted DNA evident in electrophoretic images (Figure S1).

Ionic interaction tests using modified lysis solutions containing; 1) 2 M sodium chloride, 2) 1 M ammonium sulfate, and 3) 1 M ammonium phosphate, yielded no measureable DNA or RNA from the clay environment. Concentrated sodium chloride caused problematic clay sample expansion, limiting recovery of the solution. Post lysis washes with concentrated ammonium sulfate (pH 7.0) and ammonium phosphate (pH 7.0) failed to yield NA from the stream sediments or iron cemented clay, and also blocked extraction of discoloring soil organic matter.

**Figure 2:**
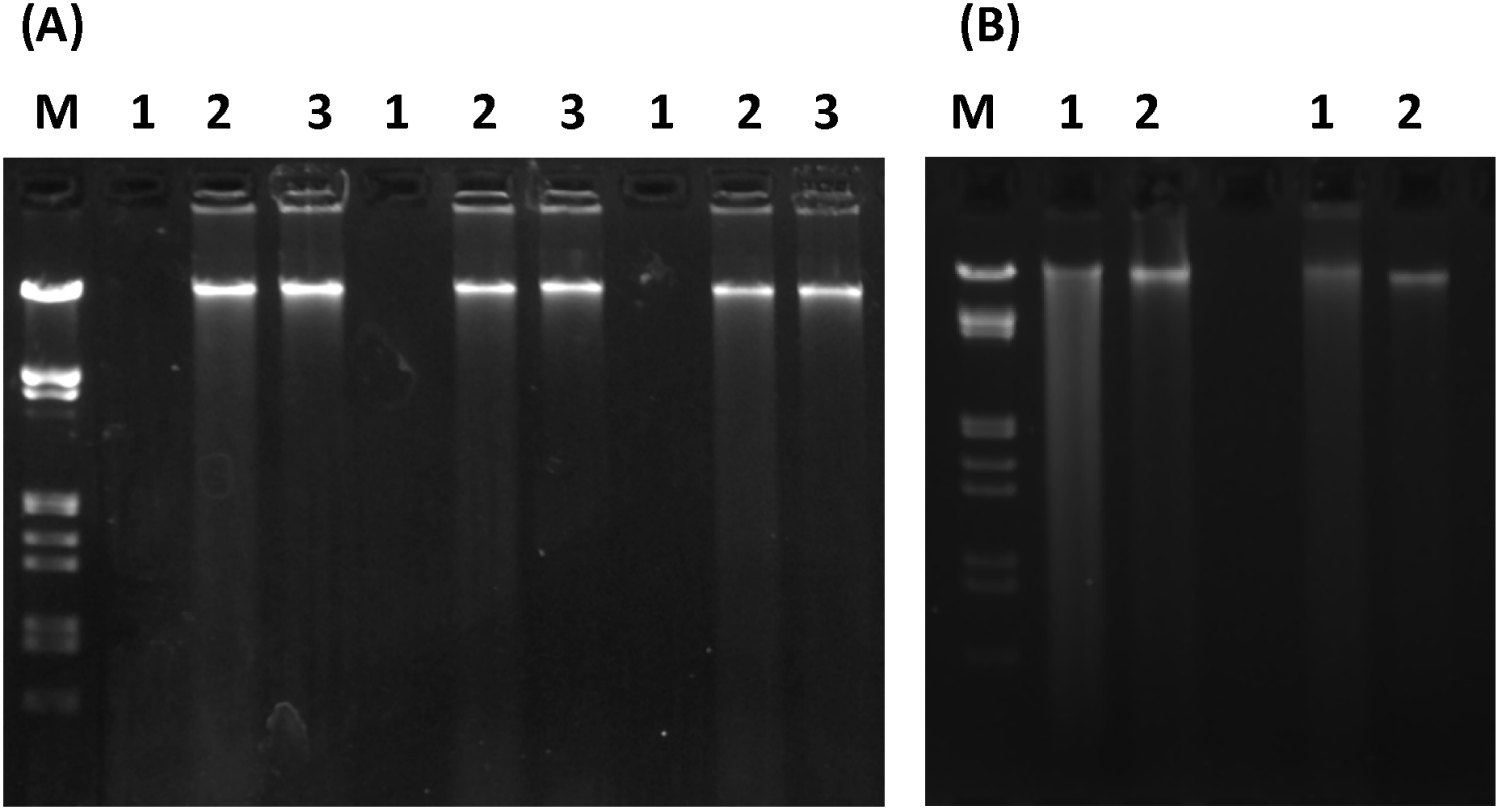
Phosphate test. (**A**) PB pellet wash test. Lysis was performed for 3 replicate extractions using (4 M guanidine thiocyanate; 100 mM MOPS (pH 7.0) 25 mM EDTA; 1% sarkosyl). Lanes 1, standard extraction process result from 200 mg iron cemented clay. Lanes 2, first of two clay pellet washes using 250 µl PB (pH 7.0). Lanes 3, second clay pellet wash using 250 µl PB. Lane M; Marker III (Roche). **B)** Test with PB in the lysis reagent. (**A**) Lane 1; extraction result from lysis reagent containing 1 M PB and 6 M guanidine HCl followed by sample dilution in a concentrated salt based extraction buffer. (**B**) Lanes; extraction result from lysis reagent containing 1 M PB and 6 M guanidine HCl followed by sample dilution in 1. 4 ml urea based extraction buffer. Lanes 2; result from 250 µl clay pellet PB wash (Both A and B). Lanes M; Marker III (Roche). Samples were electrophoresed for 90 min at 3 V/cm in 1×TAE buffer with 100 ng/ml ethidium bromide.

After the initial non-productive extraction (Figure 2A; lanes 1), HMW DNA could be desorbed from iron oxide cemented clay by suspension with a 1∶1 clay weight/1 M PB volume (500 mM Na_2_HPO_2_, 500 mM NaH_2_PO_4 _pH 7.2), followed by an additional centrifugation step (10,000× g, 1 min, 20°C; Figure 2A lanes 2). A second pellet wash was productive (Figure 2A lanes 3) while a third wash was found to offer a negligible yield (data not shown). The DNA from the stream sediment cores recovered using this PB desorption approach showed a HMW electrophoretic migration profile where the small amount of DNA visible in ethidium bromide TAE gels was highly fragmented for both LN_2_ grinding and bead mill preparative methods used without PB desorption (data not shown). By applying the phosphate desorption we extracted 1.1 µg±0.1 µg HMW DNA/gram sediment from the mile 5.0 EFPC stream sediment cores, 0.6 µg±0.1 µg HMW DNA/gram sediment from the mile 22.3 EFPC stream sediment cores, and 0.5 µg±0.0 µg HMW DNA/gram sediment from the background control site stream sediment cores, where prior to PB desorption development, the yield from all preparations using as much as 5 g of stream sediment was below A_260_ measurement capability.

Using the previous liquid N_2_ grinding method [14] to extract DNA from an iron cemented stream sediment clay environment, a concentrated salt based extraction buffer required additional steps due to phosphate precipitation with 0.6 volumes of isopropanol. The lysis reagent (1 M PB pH 8.0, 6 M guanidine HCl) yielded DNA during the initial extraction where 1.4 ml of the salt buffer (1 M NaCl, 100 mM sodium phosphate pH 8.0, 100 mM Tris⋅HCl) was used to wash the sample from the mortar (Figure 2B, lanes 1). Suspension of the clay pellet in 1 M PB (pH 7.2), followed by centrifugation, yielded additional larger DNA fragments (Figure 2B, lanes 2). This indicated the stronger adsorption of longer DNA fragments, requiring an increased phosphate concentration for desorption. A second 1 M PB wash did not yield measurable NA, suggesting that efficient extraction can be accomplished with a single phosphate buffer wash when an extraction buffer containing a high salt concentration is used in conjunction with 1 M sodium phosphate in the lysis reagent.

**Figure 3:**
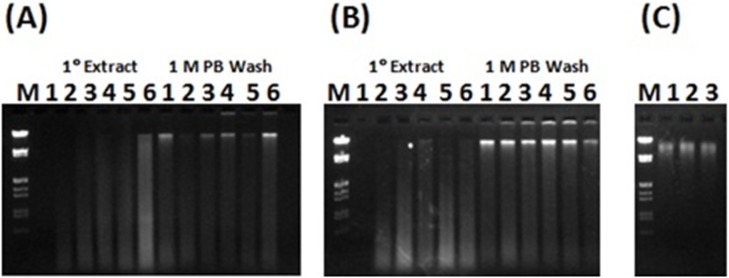
Phosphate gradient test. (**A**) 1° extract. 1.2% agarose gel using 0.1 µg/ml ethidium bromide in 1×TAE at 3 V/cm for 90 min. Lanes 1–6 (1° extract); 0, 100 mM PB, 200 mM PB, 300 mM PB, 400 mM PB, and 500 mM PB in the lysis reagent respectively. Ten percent of the total yield from the initial extraction process from 200 mg wet weight of the iron oxide cemented clay environment electrophoresed in each lane. 1 M PB Wash. Lanes 1–6 show the results of a re-wash of the sedimented clay pellets from the 1° extraction process with 1 M sodium phosphate buffer (pH 7.2). Ten percent of the total yield from the 1 M PB clay pellet re-wash was electrophoresed. Lane M; Marker III (Roche). (**B**) Second of three replicate extraction sets electrophoresed as given for (**A**) showing the extent of set to set variability. (**C**) 1.2% agarose gel using 0.1 µg/ml ethidium bromide in 1×TAE at 4.7 V/cm for 90 min. Lanes 1–3; replicate extractions from 250 mg samples of the iron oxide cemented clay using the Powerlyzer, PowerSoil® DNA isolation kit (MoBio); 10% of the total yield. Lane M; Marker III (Roche).

Extraction from iron-cemented clay yielded no measureable NA with either *TruRNA* reagents (Atom Sciences) or PowerLyzer reagents (MoBio) when extraction tests were performed on the same day the samples were recovered from their natural environment. Triplicate extractions using a gradient of PB concentrations in the lysis reagent were performed to determine the optimal TNA recovery concentration. No PB in the lysis solution resulted in no NA recovery (Figure 3A, 1° Extract, lane 1), while some DNA was obtained with 100 mM PB (Figure 3A, 1° Extract, lane 2), and as the PB concentration increased, an increase in both the extracted DNA concentration and DNA fragment length was observed (Figure 3A, 1° Extract, lanes 3–6). The amount of DNA recovered from the PB wash was similar to the re-wash of the pellet obtained with phosphate in the lysis solution, demonstrating that lysis was effective with or without phosphate in the lysis reagent (Figure 3A, 1 M PB Wash, lanes 1–6). DNA from the initial (1°) extraction procedure was kept separate from the 1 M PB wash result and both fractions were purified using a PowerClean DNA Cleanup kit (MoBio). The majority of the DNA from the PB wash was lost during purification. This is likely due to the PowerClean DNA Clean-Up kit being optimized for purification of sheared DNA resulting from bead milling procedures, and the bulk of the DNA recovered from the PB wash procedure was HMW (>20 kbp).

Even though DNA was lost during purification, the total yield was larger from the grinding process (7.96±3.03 µg/g clay). Despite a lack of DNA recovery in any of the three replicate 1° extractions that had no PB in the lysis reagent, the amount of DNA recovered from the PB wash was similar to the re-wash of the pellet obtained with phosphate in the lysis solution, demonstrating that lysis was effective with or without phosphate in the lysis reagent (Figure 3B). Even with the problematic DNA loss during purification using the PowerClean DNA Clean-Up kit, the total yield was larger from the grinding process (7.96±3.03 µg/g clay sample) than with the Powerlyzer PowerSoil® DNA isolation kit (2.46±0.25 µg/g clay sample (Figure 3C). Agarose gel purification with the Wizard DNA Clean-Up System (Promega) supported improved recovery of HMW fraction yielding 15.22±2.33 µg/gram clay.

**Figure 4:**
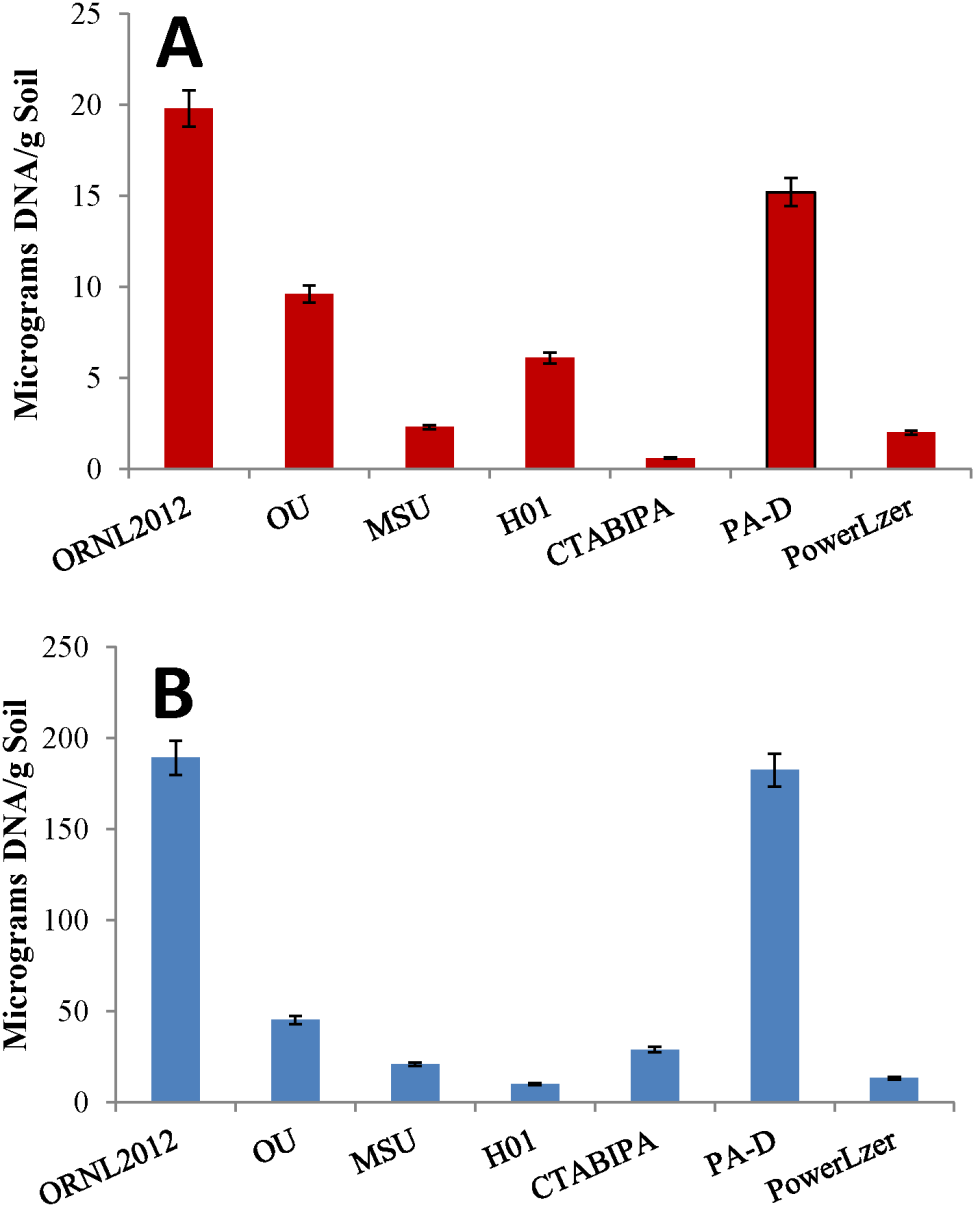
Comparison of methods. Various published and commercial procedures were compared for total DNA recovery from (**A**) clay and (**B**) A-horizon soils. Commercial kits included the Powerlyzer, PowerSoil® DNA isolation kit (MoBio) while the published methods included OU [15], MSU [39], ORNL 2001 [14], CTABIPA [40] and PA-D [37].

Additional procedures were compared for recovering HMW DNA from clay environments. Procedures that used extraction reagent heating with iron cemented clay resulted in excessive DNA fragmentation (Figure S2). The yield of DNA from alternative methods varied to a large extent on the method for final purification prior to quantification. For example, method OU and MSU were the same process followed by gel purification or use of PowerSoil DNA CleanUp reagents (MoBio), respectively. Excessive DNA fragmentation in samples heated in the presence of iron cemented clay (65°C×2 h) resulted in poor yield from agarose gel purification (OU and MSU Figure 4, Table S2) because the 1 cm gel plug only contained the HMW fraction. Moreover, binding and washing purification methods whether based on the membrane or resin based binding matrices (PowerSoil; MoBio or Wizard DNA CleanUp; Promega respectively) yielded extensive HMW DNA loss. Despite shearing, LN_2_ grinding yields larger DNA fragments than bead milling, and while the PA-D method uses a modified bead mill procedure and also resulted in a high yield of DNA from the iron cemented clay (Figure 4, Table S2) the DNA was fragmented in a manner typical of other bead milling procedures. The CTAB-IPA method failed to yield nucleic acid from the clay sample (Figure 4, Table S2), and was known not to work with ORNL IFRC samples suggesting the absorptive properties of the regional sediments was the cause. Overall, while the presently described ORNL 2012 and the PA-D methods resulted in the highest yields of DNA from clay and A-horizon soils (Table S2), ORNL 2012 was the only method to maintain HMW DNA, critical to sequencing efforts (Figure 4).

### Comparison of accessed microbial community diversity

Triplicate DNA extractions for each of the ORNL 2001 and 2012 methods, as well as the PowerLyzer™ reagents (MoBio) were amplified using pyrosequence tagged primers targeting the V1–V3 region of the 16S rRNA gene. The multiplexed amplicons were processed in batch resulting in a total of 61,674 high-quality bacterial (mean = 3855 reads; n = 16) sequences (Table 1). Any potential sequences with indels and chimeras were removed using ampliconnoise and UCHIME respectively. The raw data files (sff) for each sample have been deposited in the NCBI Sequence Read Archive under Accession No. SRA089217.

**Table 1:** Comparison of Methods by OTU Counts^a^ percentage of OTUs from Dominantly Gram Positive Phyla.

Method	Clay			A-horizon		
	OTUCount^b^	%*Firmicutes*±sd	%*Actinobacteria*±sd	OTUCount^b^	%*Firmicutes*±sd	%*Actinobacteria*±sd
ORNL 2001	233±48	0.07±0.06	11.47±2.2	521±16	0.05±0.07	15.65±0.07
ORNL 2012	495±6	1.73±1.10	7.90±1.31	568±60	1.00±0.28	11.45±3.32
PowerLyzer™	252±29	0.13±0.15	6.30±1.76	535±23	0.07±0.06	10.77±2.15

a Data is from closed-reference picked OTUs, rarefied at 1404 sequences per sample.

b Values are averages among replicate extractions.

**Figure 5:**
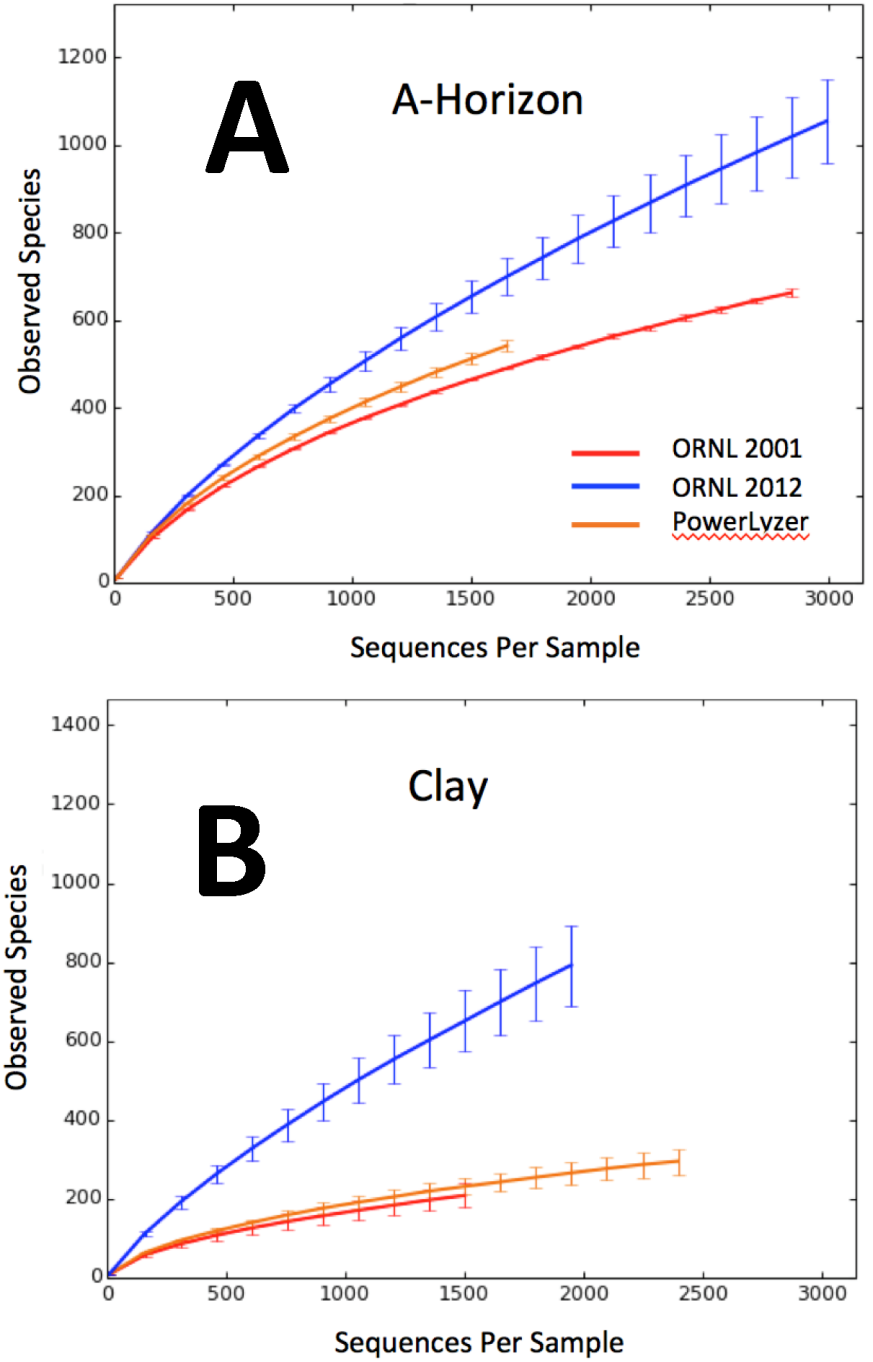
Rarefaction based comparison of Extraction Procedures. Rarefaction analysis was performed using the de-noised 454 pyrotag sequencing data on *de novo* picked OTUs for (**A**) A-horizon and (**B**) clay samples. Each curve is the average from experimental replicates (N = 2 libraries for the 2001 method and ORNL 2012 method A-horizon samples; N = 3 libraries for the remainder of the sample/method combinations) from 100 jackknife iterations over each sampling depth. The key for the A-horizon comparison of accessed diversity uses the same color code as for the clay sample comparison.

To compare the total recovered diversity among all three methods, rarefaction curves of operational taxonomic units (OTUs) clustered at 97% similarity were generated using QIIME v1.8. The averaged rarefaction curves showed that the recovered diversity was ∼1.8-fold higher with the presently described ORNL 2012 method for A-horizon soils and 4-fold higher when applied to clay (Figure 5). As a second measure of determining increased diversity recovery from sediments, we also used a “closed reference method” which assigned sequences to OTUs based upon the similarity to a well characterized sets of 16S rRNA sequences by utilizing the Greengenes database (http://greengenes.lbl.gov/cgi-bin/nph-index.cgi). Classifications were performed at both 97 and 99% sequence similarity restrictions (Figures S3, S4) and also showed similar trends of comparatively higher diversity recovery with the ORNL 2012 method. The average increase in extractable OTUs for clay samples, via the closed-reference OTU picking is shown in Table 1. Low variation among experimental replicate extractions showed that the phylodiversity accessed was reproducible for a given method (Tables 2, 3).

**Table 2:** Phylodiversity by Extraction Method.

	Method	Mean PD	Std. Dev	P-value^a^	FDR^b^ Adj.P-val
	ORNL2012	39.8953	0.9027	4.81E-06	1.44E-05
Clay	ORNL2001	20.7136	2.3867	2.01E-04	3.01E-04
	PowerLyzer	20.8968	3.1258	5.38E-04	5.38E-04
	ORNL2012	43.5448	3.4232	2.87E-02	8.62E-02
A-Horizon	ORNL2001	36.8989	1.9681	1.40E-01	1.40E-01
	PowerLyzer	38.9997	0.8721	9.85E-02	1.40E-01

a P-values denote comparisons of the ORNL2012 vs corresponding row Method. The p-value for the ORNL2012 row represents ORNL2012 vs the other two methods combined.

b FDR = false discovery rate.

**Table 3:** Phylodiversity by Extraction Method for Selected Phyla.

		Method	Mean PD	Std. Dev	P-value^a^	FDR Adj.P-val
***Clay***	***Firmicutes***	ORNL2012	2.329	0.6147	0.0034	0.0101
		ORNL2001	0.666	0.5855	0.0275	0.0275
		PowerLyzer	0.647	0.5624	0.0250	0.0275
***Clay***	***Actino bacteria***	ORNL2012	3.734	0.0958	0.0057	0.0086
		ORNL2001	2.893	0.5826	0.0693	0.0693
		PowerLyzer	2.415	0.1010	0.0001	0.0002
***A-horizon***	***Firmicutes***	ORNL2012	1.850	0.6663	0.0411	0.1233
		ORNL2001	0.550	0.7784	0.2147	0.2147
		PowerLyzer	0.549	0.4114	0.0913	0.1370
***A-horizon***	***Actino bacteria***	ORNL2012	4.840	0.5464	0.8689	0.8689
		ORNL2001	5.142	0.1075	0.5229	0.8532
		PowerLyzer	4.531	0.4105	0.5688	0.8532

a P-values denote comparisons of ORNL2012 vs corresponding row Method. The p-value for the ORNL2012 row represents ORNL2012 vs the other two methods combined.

**Figure 6:**
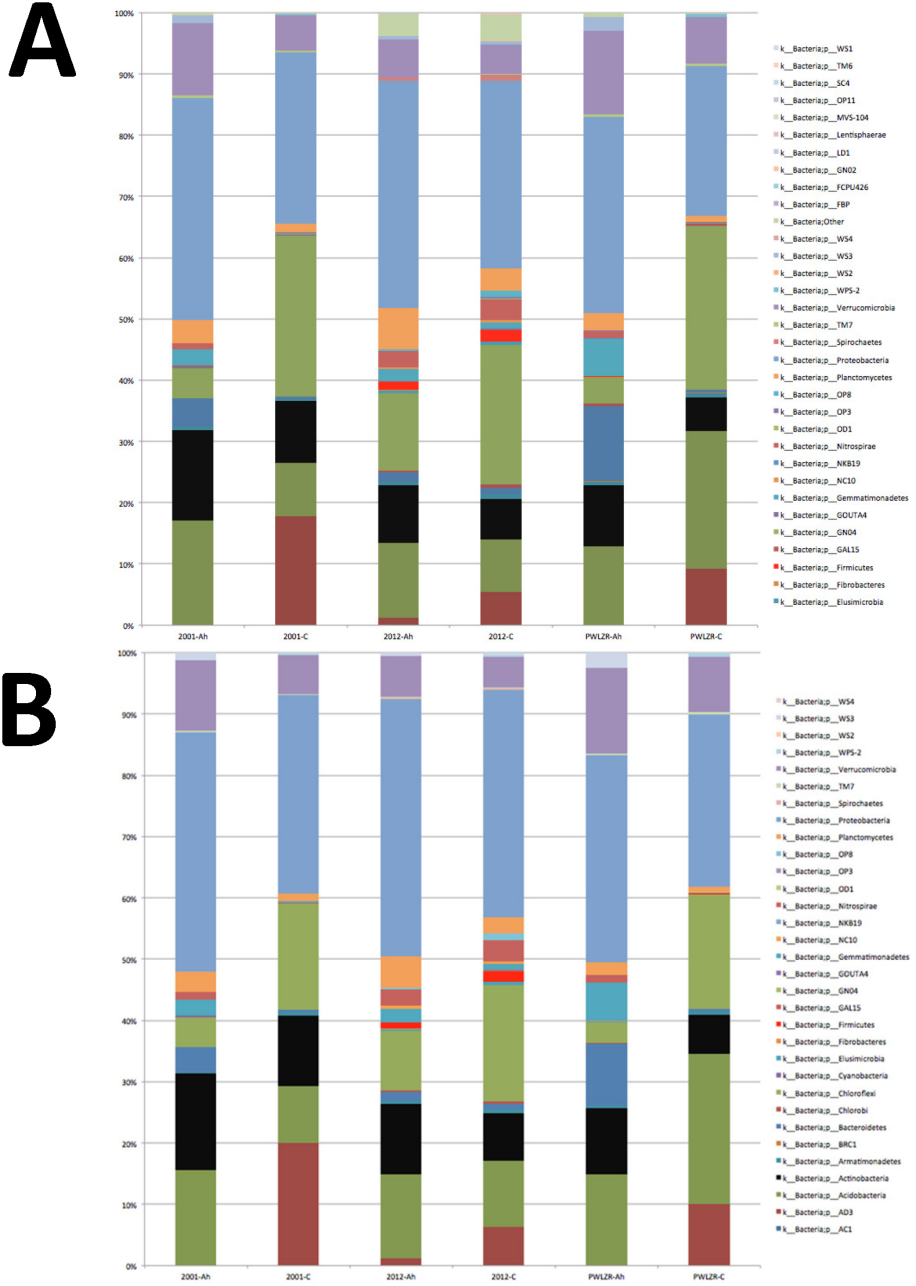
16S V1–V3 Taxonomic Summary. Phylum-level taxonomic composition of (**A**) *de novo* and (**B**) closed-reference picked OTUs rarefied at 1499 and 1404 sequences per sample, respectively. For (**A**), taxonomy was assigned to the de-noised 454 pyrotag sequencing data using the RDP Classifier [53] using a confidence threshold of 80%. Each lane is the result of summarizing the experimental replicates by extraction method (N = 2 libraries for the 2001 method and ORNL 2012 method A-horizon samples; N = 3 libraries for the remainder of the sample/method combinations). For (**B**) if a given sequence did not match within 3% of any sequence within the Greengenes database [34], [35], the sequence was discarded from the taxonomic summary. Taxonomy was assigned to the de-noised 454 pyrotag sequencing data using the RDP Classifier [53] via QIIME using a confidence threshold of 80%. Each lane is the result of summarizing the experimental replicates by extraction method. C = clay, Ah = A Horizon; PWLZR represents the samples extracted using the PowerLyzer System (MoBio).

The *Firmicutes* showed considerably (>300%) higher phylodiversity and were a higher proportion of the microbial community in the clay libraries derived from the ORNL 2012 procedure compared to the other methods (Table 3). This larger representation of *Firmicutes* was also apparent in the increased diversity from the A-horizon where adsorption is less of an issue. This large difference in diversity was not observed for the *Actinobacteria* in the ORNL2012 A-horizon libraries, but the *Actinobacteria* were 27–54% higher in the ORNL2012 clay libraries (Table 3). The ORNL 2012 method also yielded the largest number of unclassified OTUs whether using QIIME (Figure 6A) or the closed reference method (Figure 6B). The improved capacity to recover DNA from the *Firmicutes* suggests that the higher number of unclassified OTUs in clay and A-horizon libraries from the ORNL 2012 method may result from accessing bacterial species that are more difficult to lyse since the commercial and earlier methods did not capture DNA from these organisms.

**Figure 7:**
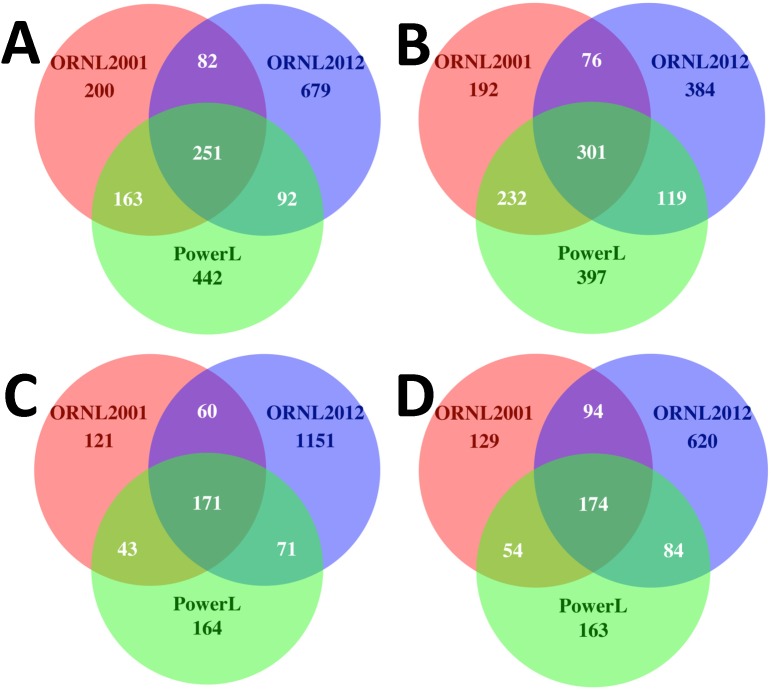
OTU Overlap among DNA Extraction Methods. Venn diagrams representing the combined OTUs accessed using experimental replicate extractions from (**A, B**) deciduous forest A-horizon soil and (**C, D**) iron oxide cemented clay. (**A, C**) The OTUs were identified and clustered using the *de novo* uclust implementation in QIIME [48] and each sample was rarified to 1499 sequences at 97% sequence similarity from sequences that were de-noised and singletons removed. (**B, D**) Venn diagram of overlapping OTUs across different methods using the *closed reference* OTU picking method with 97% sequence similarity against manually curated Greengenes database of 16S rRNA sequences.

Venn diagrams using de-noised data with singletons removed and rarified to 1499 sequences per sample showed a higher proportion of non-overlapping OTUs were recovered using the ORNL 2012 method (Figure 7A–D), suggesting improved coverage of the extant microbial community. For example, from the clay environment, 65.9% of the OTUs recovered using ORNL 2012 method were unique to the method, whereas <10% non-over-lapping OTUs were recovered using both ORNL 2001 and PowerLyzer procedure (Figure 7C). However, in A horizon soil, OTUs from the PowerLyzer kit also recovered a high proportion of non-overlapping OTUs, comparable to ORNL 2012 method (Figure 7A). Furthermore, when we only included the OTUs that had similarity in the manually curated Greengenes database of 16S rRNA sequences, i.e. OTUs picked using the closed reference method, a higher diversity was still recovered using the ORNL 2012 method even though the number of OTUs changed (Figures 7B, D).

## Discussion

Iron cemented clays are notoriously difficult to extract NA from in high molecular weights amenable to sequencing efforts. The key to overcoming this issue was minimizing adsorption and maximizing desorption from the clay particles. Competitive desorption relying on successive post-lysis washes using concentrated PB supported DNA preparation at a sufficient quantity and quality to support in-depth analysis of the extant microbial community.

TNA recovery from iron oxide cemented clay taken from subsurface stream environments was weak with all tested methods PB (>600 mM) used during grinding supported >100-fold improvement in DNA recovery from iron-cemented clay stream sediments, whereas all other efforts failed to yield measureable NA The PowerSoil kit yielded below detection results while prior LN_2_ grinding methods also demonstrated poor yields. Moreover, the subsurface stream sediment samples consistently yielded DNA exhibiting a degradation profile in electrophoretic images. The stream sediment samples had a strong hydrogen sulfide odor that likely caused radical-associated damage after lysis [21].

Prior work based on hot SDS lysis methods that include phosphate in the extraction buffer such as the OU and MSU methods [15] yielded DNA from the terrestrial iron cemented clay, however the DNA was heavily damaged despite inclusion of 50 mM EDTA (Figure S2). This is likely a result of a Fenton type reaction since pyrite induces DNA damage by converting water to H_2_O_2_, and iron oxides react with H_2_O_2_ to produce hydroxyl radicals that rapidly degrade NA [22]. For the OU method, the low yield was due to little DNA remaining in the 1 cm plug cut from the HMW region.

For iron cemented clay, extraction reagent pH adjustment did not improve NA yield. Humic acids share many properties with NA and exhibit stronger adsorption to clay minerals and iron oxide coated surfaces at a lower pH [6], [23], [24]. Moreover, DNA adsorption to soil consisting largely of montmorillonite and illite silt was blocked at pH 10.0, and NA adsorption to cation cemented montmorillonite decreased as the pH increased to ∼6.0 [25]. Ogram *et al.* recovered NA from subsurface clay, but the effort and amount of material required (in the kilogram range) is impractical. For LN_2_ grinding, adsorption likely occurs during grinding when the NA is in direct contact with adsorptive particles.

The finding that DNA desorption from iron cemented clay with 1M PB was in a higher molecular weight range than DNA recovered from with lower phosphate concentrations is in agreement with humic acid adsorption studies [6], [13], [26], but contrasts with length dependent measurements of DNA adsorption to soils [7]. Ogram *et al.* demonstrated that smaller DNA fragments have faster adsorption kinetics [27]. While smaller fragments may bind faster due to a larger diffusion coefficient [28], they are also more readily desorbed [5], and the present work shows that larger DNA fragments adsorb more strongly, presumably because of the increased number of contact points. The rate of NA adsorption and reversibility of NA adsorption are therefore separate aspects affecting the end product of an extraction process.

There has been little effort directed to comparison of DNA extraction procedures based on community 16S rRNA studies [29]. Four plausible explanations for the elevated diversity observed from clay with the ORNL 2012 procedure include; 1) improved cell lysis, 2) artificial diversity from DNA damage, 3) diversity accumulation from DNA sequestration and protection against nucleases by adsorption, and 4) sampling variation among replicates. First, the ORNL 2012 lysis procedure avoids oxidative DNA damage from heating or storage in the presence of humic acids or materials with a high iron or sulfide content [22], [30], [31]. Secondly, the elevated diversity using the ORNL 2012 procedure was also evident in the A-horizon pyrosequencing libraries where the phosphate desorption procedure was not applied. The increase in biodiversity coincides with an increase in recovered DNA quantity. To our knowledge there is no known mechanism for selective adsorption of DNA from different groups of organisms. It is possible that Gram positive organisms lyse later in the grinding process when most of the clay binding capacity is occupied. Moreover, it is also unlikely that artificial diversity from DNA damage inflated the number of OTUs in ORNL 2012 methods as our results – higher number of non-over-lapping OTUs obtained from ORNL 2012 method – were consistent with the more stringent “closed-reference OTU picking” strategy of QIIME [32] where only OTUs that match sequences present within the curated Greengenes database were retained (Figures 7, S3–S5) [33], [34], [35]. This method assesses the diversity of known microorganisms within a set of samples. We used the “closed-reference” approach to only retain OTUs that are 97% or 99% similar to full-length curated sequences that have already been verified in other studies. Although the closed-reference approach results in a dramatically reduced number of total OTUs, compared to *de novo* OTU-picking, the overall pattern of increased OTU diversity using the ORNL2012 protocol remained (Tables 1, 2, Figures 5, S3, & S4). Finally, because all extractions for pyrosequencing were performed on the same day from the same sample stock, and strong reproducibility was observed within replicates, sampling variation is an unlikely source of the observed increase in diversity.

Comparisons of the adsorptive behavior of RNA and DNA to mineral particulates have demonstrated a difference that is likely related to stranded-ness [28], [36], [37]. This differential adsorption of DNA and RNA varies among environmental matrices and requires normalization when the goal of co-extraction of TNA is gene expression/gene dosage evaluations. For our experiments, inclusion of 1% sarkosyl and BSA in the lysis solution resulted in improved DNA quantity implicating additional adsorption mechanics other than ionic binding.

Use of concentrated phosphate supports a consistent and efficient extraction of HMW DNA and RNA simultaneously from clay containing high levels of iron. DNA of this quality along with the corresponding mRNA should be especially useful for systems biology efforts including microbial community genomic and transcriptomic studies. This is a substantial step forward in obtaining high quality nucleic acids from microbial communities that have historically been difficult or impossible to obtain. Information gained from these newly accessible organisms is likely to help in unraveling the structure-function relationships within microbial communities since community members of most, if not all, environments can now be identified.

## Experimental Procedures

### Collection of, soil, and sediment samples

Three stream sediment core samples were taken from kilometers 5 and 22.3 of East Fork Poplar Creek (EFPC) as well as Hinds Creek, Oak Ridge TN, at a depth of 45 cm. Samples were collected with the permission of the US Department of Energy under the existing agreement with Oak Ridge National Laboratory and the ORNL Hg Science Focus Area. These field studies did not at any time involve endangered or protected species. The cores were frozen in the field in LN_2_ and stored at −80°C until processed. These cores were heterogeneous in texture and consisted largely of gravel, sand, coal fragments, and iron cemented clay. To prevent loss of precious sediment, alternative samples containing iron cemented clay were used for method development. A clay sample was taken at 20 cm below the surface of deciduous forest A-horizon in Knoxville, TN and maintained at 21°C for >3 days. The top 5.1 cm of A-horizon soil were used to evaluate the reagents and ensure that the DNA and RNA were protected throughout the extraction process. The iron cemented clay had a uniform appearance, and extractions were performed on 250 mg samples that were taken from a larger piece that had the exterior portion cut away using a sterile spatula. A-horizon samples were placed in a plastic bag, root and other material present in larger pieces were removed, and the residual soil was manually mixed to prepare a uniform loamy material.

### Carbon, Nitrogen and Iron content determination

All samples including the deciduous forest surface and sub-surface soil samples used for process development were determined for total organic carbon and nitrogen content by combusting about 0.2 g soil at 950°C using a LECO TruSpec CN Determinator 630 (LECO Co, MI) (Table S1). Acid extractable iron content was determined by suspending 0.5 g (dry weight) soil or sediment in 5 ml of 0.5 M HCl shaking vigorously for 4 h, followed by centrifugation at 60×g for 15 min to obtain the clear supernatant (or acid extract). In addition, the total iron content was determined by digesting 0.5 g soil or sediment (dry weight) in 1 ml concentrated HNO_3_ (67–70%) and 0.5 ml concentrated HCl (37.9%) at 95°C for 4 hours, followed by dilution with deionized water to a final volume of 5 mL [38]. Total metal content in the acid extracts and digests was determined, after appropriate dilutions (10–100x), using an inductively coupled plasma mass spectrometer (ICP-MS, Elan-DRC, PerkinElmer, CT). Working standards were prepared in the range from 0.1 to 10 mg/L in dilute HNO_3_ (0.01 M).

### Nucleic acid extraction process and modification

Frozen stream sediment samples, or live room temperature clay and A-horizon samples used for process development, were loaded into sterile mortars and overlaid with lysis solution (4 M guanidine isothiocyanate; 100 mM Tris pH 7.0, 20 mM EDTA, 0.6% sarkosyl, 10 µl/ml 2-mercaptoethanol) or clay lysis solution (1 M phosphate buffer (pH 7.2), 4 M guanidine isothiocyanate, 10 µl/ml 2-mercaptoethanol) at a volume of 0.33 ml/g sample at 21°C. Lysis solution modifications including addition of PB (see below) at final concentrations ranging from 100 mM to 1 M, removal of the 0.6% sarkosyl, and inclusion of 10% w/v BSA were tested. Samples were dispersed for <5 seconds with a pestle and overlaid with liquid nitrogen (LN_2_), ground until thawed twice, and washed from the mortar into centrifuge tubes with concentrated urea extraction buffer (4.67 ml/g sample; 6.4 M urea, 1 M NaCl, 100 mM MOPS (pH 7.0), 40 mM EDTA). Extraction solutions were centrifuged (4500×g, 10 min, 21°C) in a bench top centrifuge (large scale preparations), or in a micro-centrifuge (10,000×g, 1 min, 21°C; small scale preparations) depending on the sample quantity. A detergent mixture (5% sarkosyl and 5% cetyltrimethylammonium bromide) was added (1.3 ml/g), followed by 5 M potassium acetate (0.67 ml/g, pH 5.2). The resulting solutions were extracted by vigorous mixing with an equal volume of chloroform:isoamyl alcohol (49∶1) and centrifugation (7,100×g, 20 min, 21°C). Total NA were precipitated with isopropyl alcohol (0.67 volumes), centrifuged (16,000×g, 20 min, 21°C), and dissolved in diethylpyrocarbonate (DEPC) treated ddH_2_O. A concentrated salt based extraction buffer (100 mM Tris; 1 M NaCl; 100 mM NaH_2_PO_4_, 20 mM EDTA, 1% SDS) was compared with urea extraction buffer.

For samples containing a large quantity of clay particles, the pellets were re-suspended in 1 ml/g PB [500 mM Na_2_HPO_4,_ 500 mM NaH_2_PO_4_ (pH 7.2)] by vigorous agitation or brief vortex mixing and centrifuged at 10,000×g for 1 min. The solution was transferred to a new centrifuge tube, diluted with 2.33 ml/g extraction buffer and handled as described for the initial extraction. NA was precipitated using 0.67 volumes of isopropyl alcohol (21°C, 20 min) and centrifuged (16,000×g, 20 min at 21°C). Precipitates were dissolved in DEPC treated ddH_2_O (100 µl/g). Humic acid removal was performed using a 1 cm column of Sephadex G-75 molecular exclusion resin (GE Healthcare) supplemented with 0.01 volumes of DEAE Sepharose (Sigma) diluted 1∶10 in 500 mM potassium acetate. The column flow-through was collected and where visible discoloration was present, samples were further purified using solutions 1–4 of a MoBio DNA cleanup system. The HMW DNA fraction was desorbed from the final organic matter precipitate by re-suspension in 200 µl 60°C DEPC treated ddH_2_O by repetitive pipetting. Flow charts for the phosphate buffer desorption process (Figure S5A) and workflow comparisons of the ORNL2001 and ORNL 2012 processes (Figure S5B) are supplied.

Three commercial reagent systems for extraction of TNA (*TruRNA* Atom Sciences, Oak Ridge) and RNA/DNA (RNA PowerSoil, and Powerlyzer; MoBio Laboratories, Salona Beach, CA) were compared for NA recovery from the soil sediment and clay samples. Additionally, selected published methods were compared including the methods described herein as OU [15], MSU [39], ORNL 2001 [14], CTABIPA [40] and PA-D [37]. All purchased reagent systems were used according to the manufacturer’s instructions as of June, 2012. The reason for using the MoBio kits as the standard for comparison of the selected published procedures as well as the presently presented work is that it is not only a commercially available kit but apparently a popular one. Use of this kit as the standard would more readily allow other laboratories to compare their results against the kit and the presently presented work.

### Extraction process dependence of microbial community composition

Three methods that supported DNA recovery from deciduous forest iron-cemented clay and A-horizon soil were compared on the basis of recovered microbial diversity in replicate DNA extractions. All extractions of DNA from clay and A-horizon samples used for comparison of accessed microbial communities were performed on the same day. Community structure was evaluated by quantification of the bacterial 16S rRNA genes using the V1–V3 region for pyrosequencing with the Titanium 454 chemistry [41], [42], [43], [44]. The 16S rRNA gene specific region of the PCR primers FLXA_27F 5′–cgt atc gcc tcc ctc gcg cca tca gAG AGT TTG ATC CTG GCT CAG–3′ and FLX_B534R 5′–cta tgc gcc ttg cca gcc cgc tca g**cg caa c**TY ACC GCG GCT GCT GG −3′ is shown in uppercase with the sequence tag identifying the sample shown in bold for one of the reverse primers. These specific primer sequences and the V1–V3 region of the 16S rRNA gene were chosen for this study as they have been shown to best represent the overall composition of synthetic communities among primers designed to amplify several different regions of the 16S rRNA gene and displayed lowest variability among replicate experiments [45]. Primary amplification used 10 ng of purified DNA (2.15×10^6^ *Escherichia coli* genome equivalents) with Platinum® *Taq* DNA polymerase High Fidelity (Invitrogen) for 27 dissociation cycles at 95°C×30 s, annealing at 55°C×30 s, and extension at 68°C×30 s. The amplification products were evaluated by electrophoresis, purified using magnetic particle separation (AgenCourt AMPureXP, Beckman Coulter) and re-evaluated for concentration and purity using an Agilent 2100 BioAnalyzer (Agilent Technologies, Inc. Santa Clara, CA) and Pico-Green (Promega, Sunnyvale, CA) in a Synergy MX Multi-Mode Reader (BioTek). The V1–V3 amplicons were then sequenced using a 454 Life Sciences Titanium gene sequencer system according to manufacturer instructions (454 Life Sciences-Roche, Branford, CT). All compared amplification products were multiplexed onto a single 454 pyrosequencing run. Two libraries amplified from A-Horizon soil samples failed to produce a comparable number of sequences in the multiplexed pyrosequencing file and were discarded.

Pyrosequencing errors [46] were removed using the AmpliconNoise algorithm [47]. The high quality sequences were binned based on barcodes using QIIME [48] and the sequences were aligned against the bacterial SILVA database and trimmed to the same length using mothur v1.22.1 [49]. Chimeric sequences were flagged and removed using UCHIME in Mothur [50].

### Clustering, taxonomic classification, and statistical analysis

Sequencing data was processed via two OTU-picking strategies via QIIME [32]: *open-referen*ce (i.e. *de novo*) and *closed-reference*. The difference being that the *closed-reference* approach only retains OTUs that have been observed within the curated GreenGenes database [34], [35].

For the *de novo* approach, sequences were assigned to OTUs at 3% distance using the uclust implementation in QIIME [48] and each sample was rarified to 1499 sequences after removal of singletons (OTUs identified only once across all samples) to minimize the number of spurious OTUs [51], [52]. Here, we removed any potential sequences with indels and chimeras using ampliconnoise and UCHIME respectively [47], [50]. A representative sequence from each OTU was selected and taxonomically identified using the Ribosomal Database Project (RDP) Classifier with a confidence cutoff of 0.8 [53], [54] via QIIME [48]. Overlapping OTUs were calculated across replicates of the three compared extraction methods and across A-horizon and clay samples using the Venn Diagram package in R statistical software [55]. Singletons were removed to support a comparison with prior work that examined the utility of pyrosequencing-based taxon identification microbial community determination [51]. Rarefaction curves and overlapping OTUs between samples were calculated after removing singletons using QIIME v1.8 [48].

The *closed-reference* approach was used to confirm that the increase in detected community sequence diversity was not due to erroneous base modification of our extraction protocol. This approach retains only the sequence data that match the full-length curated sequences contained within the Greengenes reference database [34], [35]. This *closed-reference* approach was used at two stringency levels, 97 and 99% sequence similarity. That is, if a given sequence in the data did not match within 1 or 3% of the curated sequences within the Greengenes database, the sequence was discarded. All other processing was identical to the *de novo* approach.

## Supporting Information

Figure S1 **Evaluation of lysis reagent additives.** Panels (A), (B), and (C) are electrophoretic images taken from independent extraction experiments done in triplicate. Electrophoretic images are from a single 1.2% agarose gel using 0.1 µg/ml ethidium bromide in 1×TAE at 3 Vcm^−1^ for 90 min. Lanes 1, 3, and 5 are 5% of the total direct extraction product DNA yield from 200 mg iron cemented clay (wet weight) performed using 0.33 volume of the modified lysis reagents. Lanes 2, 4, and 6 show the results from the PB wash procedure done for the extracted clay samples pellets remaining after extraction shown in lanes 1, 3, and 5 respectively. Lanes 1 used lysis reagent containing 300 mM PB added to the standard lysis reagent (see methods) with sarkosyl omitted. Lanes 3 show the result from standard lysis reagent supplemented with 300 mM PB and 1% sarkosyl. Lanes 5 show the result from standard lysis reagent supplemented with 300 mM PB, 1% sarkosyl, and 10% w/v BSA.
(DOCX)

Figure S2 **Comparison of LN_2_ Grinding Procedures.** (A) Preparative electrophoresis gel containing triplicate extractions using the ORNL 2012 procedure from deciduous forest subsurface (Clay) and surface (A-horizon) samples. Lanes marked 1 contain 50 µl of 200 µl l primary extract from 250 mg soil. Lanes marked 2 contain 50 µl of 200 µl phosphate desorption product from 250 mg soil. (B) Preparative electrophoresis gel containing NA extracted using the OU procedure (Figure S3) from deciduous forest subsurface (Clay) and surface (A-horizon) samples. Lanes marked 1, 2, and 3 contain 50 µl of 200 µl NA extract from triplicate 5 g soil samples.
(DOCX)

Figure S3 **Closed-reference rarefaction curves based on 97% sequence similarity.** Rarefaction curves based on 100 jackknife iterations at each sampling depth for OTU’s picked at 97% sequence similarity via closed-reference OTU picking against the Greengenes database [34,35,56]. If a given sequence did not match within 3% of any sequence within the Greengenes database the sequence was discarded from the analysis.
(DOCX)

Figure S4 **Closed-reference rarefaction curves based on 99% sequence similarity.** Rarefaction curves based on 100 jackknife iterations at each sampling depth for OTUs picked at 99% sequence similarity via closed-reference OTU picking against the Greengenes database [34,35,56]. If a given sequence did not match within 1% of any sequence within the Greengenes database the sequence was discarded from the analysis.
(DOCX)

Figure S5 **Workflow diagrams.** (A) The step-wise workflow of the phosphate buffer desportion process to recover DNA that may be bound to particulates. (B) A comparative workflow highlighting the differences between the ORNL2001 and ORNL2012 processes for obtaining high molecular weight DNA.
(DOCX)

Table S1 **Basic sample chemistry.**
(DOCX)

Table S2 **Extraction method comparison of DNA Yields.**
(DOCX)
