# Supplementary material for: Improved Yield of High Molecular Weight DNA Coincides with Increased Microbial Diversity Access from Iron Oxide Cemented Sub-Surface Clay Environments
Source: PLoS One. 2014 Jul 17;9(7):e102826. doi: 10.1371/journal.pone.0102826 (PMC4102596; doi:10.1371/journal.pone.0102826)
Supplement: Figure S1 — Evaluation of lysis reagent additives. Panels (A), (B), and (C) are electrophoretic images taken from independent extraction experiments done in triplicate. Electrophoretic images are from a single 1.2% agarose gel using 0.1 µg/ml ethidium bromide in 1×TAE at 3 Vcm−1 for 90 min. Lanes 1, 3, and 5 are 5% of the total direct extraction product DNA yield from 200 mg iron cemented clay (wet weight) performed using 0.33 volume of the modified lysis reagents. Lanes 2, 4, and 6 show the results from the PB wash procedure done for the extracted clay samples pellets remaining after extraction shown in lanes 1, 3, and 5 respectively. Lanes 1 used lysis reagent containing 300 mM PB added to the standard lysis reagent (see methods) with sarkosyl omitted. Lanes 3 show the result from standard lysis reagent supplemented with 300 mM PB and 1% sarkosyl. Lanes 5 show the result from standard lysis reagent supplemented with 300 mM PB, 1% sarkosyl, and 10% w/v BSA. (DOCX) [file pone.0102826.s001.docx]

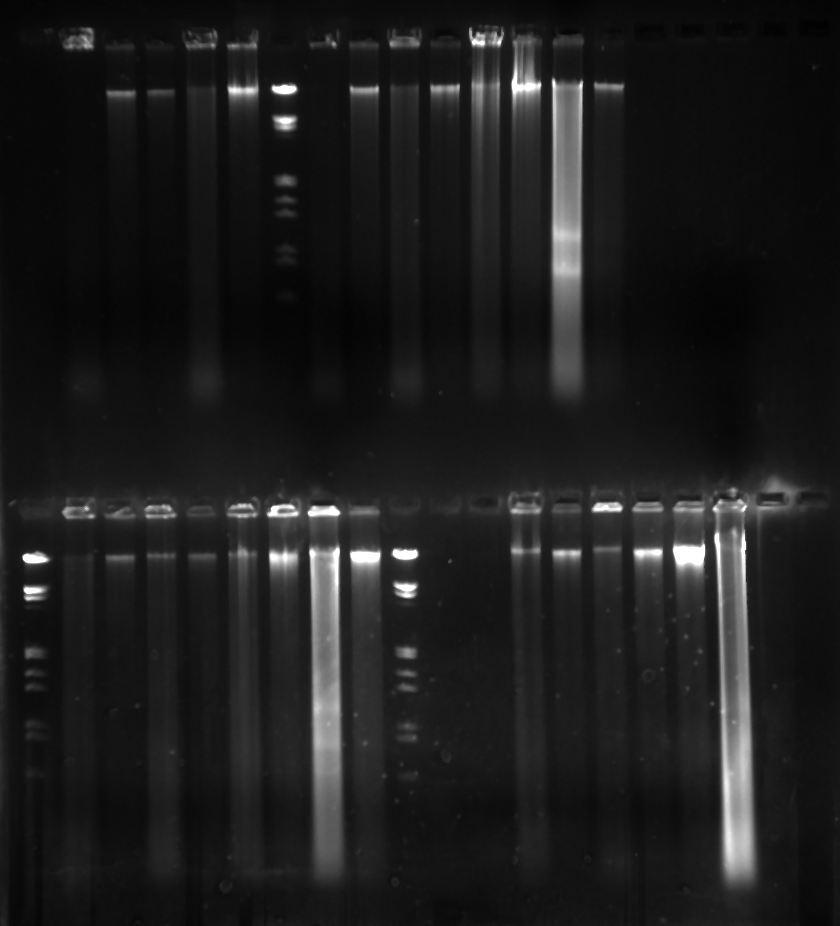

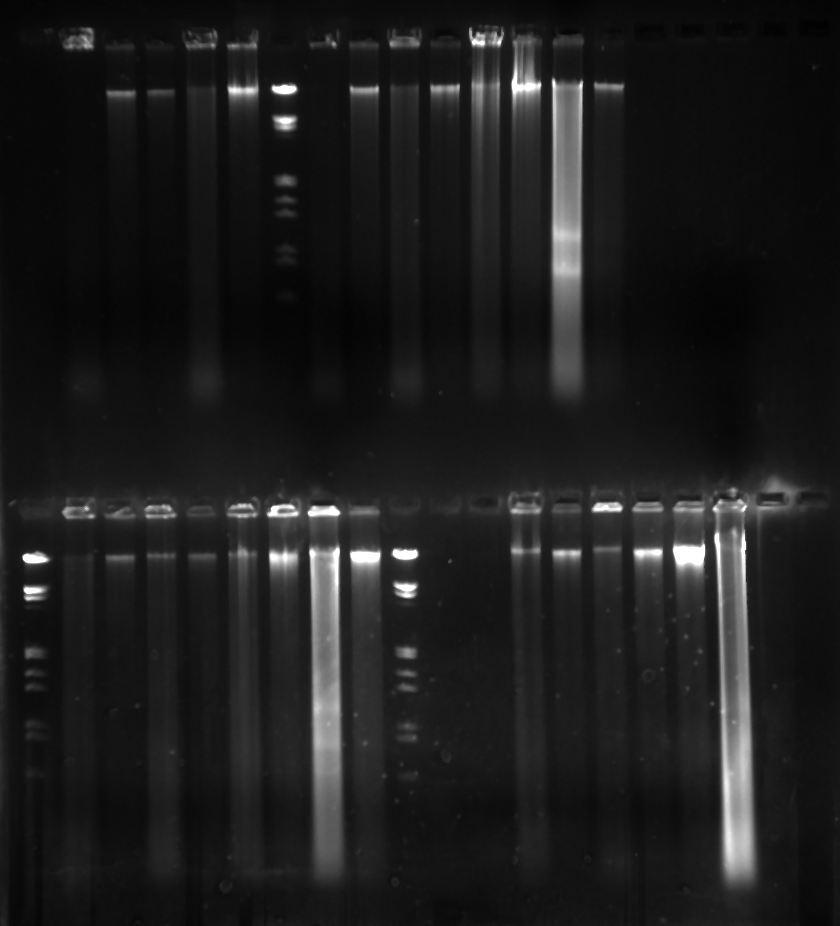

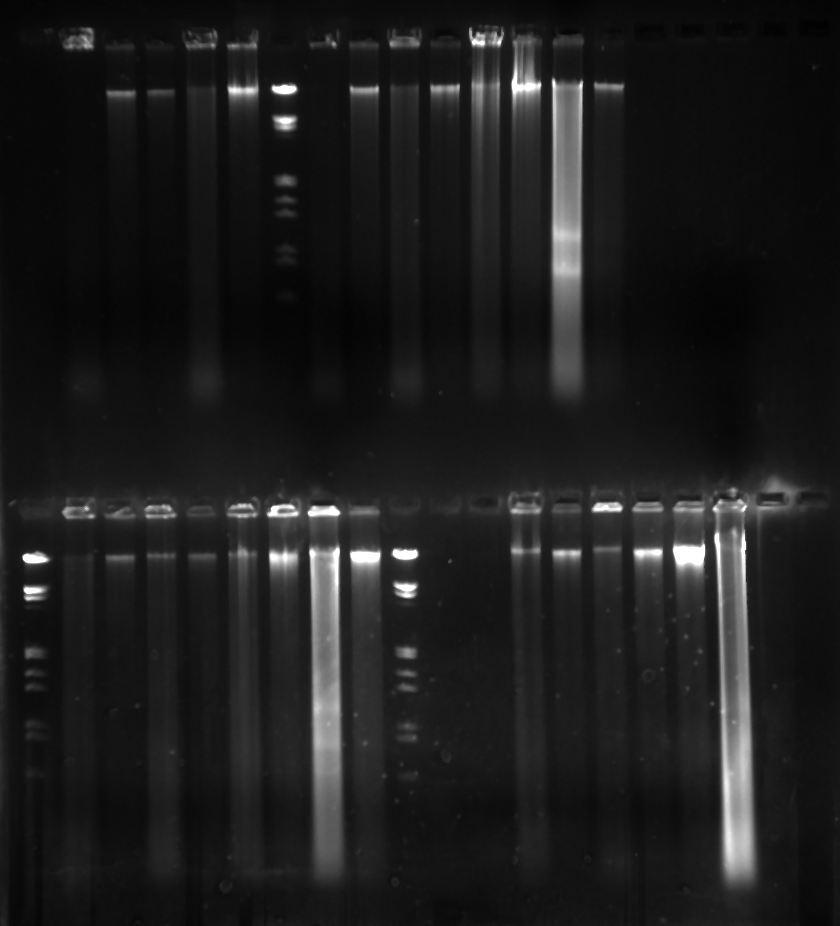


**M 1 2 3 4 5 6 M 1 2 3 4 5 6 M 1 2 3 4 5 6**

**(A) (B)**   **(C)**

**Figure S1**. **Evaluation of lysis reagent additives.** Panels (A), (B), and (C) are electrophoretic images taken from independent extraction experiments done in triplicate. Electrophoretic images are from a single 1.2% agarose gel using 0.1 µg/ml ethidium bromide in 1 × TAE at 3 Vcm^−1^ for 90 min. Lanes 1, 3, and 5 are 5% of the total direct extraction product DNA yield from 200 mg iron cemented clay (wet weight) performed using 0.33 volume of the modified lysis reagents. Lanes 2, 4, and 6 show the results from the PB wash procedure done for the extracted clay samples pellets remaining after extraction shown in lanes 1, 3, and 5 respectively. Lanes 1 used lysis reagent containing 300 mM PB added to the standard lysis reagent (see methods) with sarkosyl omitted. Lanes 3 show the result from standard lysis reagent supplemented with 300 mM PB and 1% sarkosyl. Lanes 5 show the result from standard lysis reagent supplemented with 300 mM PB, 1% sarkosyl, and 10% w/v BSA.
