# Supplementary material for: Improved Yield of High Molecular Weight DNA Coincides with Increased Microbial Diversity Access from Iron Oxide Cemented Sub-Surface Clay Environments
Source: PLoS One. 2014 Jul 17;9(7):e102826. doi: 10.1371/journal.pone.0102826 (PMC4102596; doi:10.1371/journal.pone.0102826)
Supplement: Figure S2 — Comparison of LN2 Grinding Procedures. (A) Preparative electrophoresis gel containing triplicate extractions using the ORNL 2012 procedure from deciduous forest subsurface (Clay) and surface (A-horizon) samples. Lanes marked 1 contain 50 µl of 200 µl l primary extract from 250 mg soil. Lanes marked 2 contain 50 µl of 200 µl phosphate desorption product from 250 mg soil. (B) Preparative electrophoresis gel containing NA extracted using the OU procedure (Figure S3) from deciduous forest subsurface (Clay) and surface (A-horizon) samples. Lanes marked 1, 2, and 3 contain 50 µl of 200 µl NA extract from triplicate 5 g soil samples. (DOCX) [file pone.0102826.s002.docx]

**Figure S2**. **Comparison of LN_2_ Grinding Procedures**. (A) Preparative electrophoresis gel containing triplicate extractions using the ORNL 2012 procedure from deciduous forest subsurface (Clay) and surface (A-horizon) samples. Lanes marked 1 contain 50 µl of 200 µl primary extract from 250 mg soil. Lanes marked 2 contain 50 µl of 200 µl phosphate desorption product from 250 mg soil. **(B)** Preparative electrophoresis gel containing NA extracted using the OU procedure (Figure S3) from deciduous forest subsurface (Clay) and surface (A-horizon) samples. Lanes marked 1, 2, and 3 contain 50 µl of 200 µl NA extract from triplicate 5 g soil samples.
