# Supplementary material for: Improved Yield of High Molecular Weight DNA Coincides with Increased Microbial Diversity Access from Iron Oxide Cemented Sub-Surface Clay Environments
Source: PLoS One. 2014 Jul 17;9(7):e102826. doi: 10.1371/journal.pone.0102826 (PMC4102596; doi:10.1371/journal.pone.0102826)
Supplement: Figure S4 — Closed-reference rarefaction curves based on 99% sequence similarity. Rarefaction curves based on 100 jackknife iterations at each sampling depth for OTUs picked at 99% sequence similarity via closed-reference OTU picking against the Greengenes database [34,35,56]. If a given sequence did not match within 1% of any sequence within the Greengenes database the sequence was discarded from the analysis. (DOCX) [file pone.0102826.s004.docx]

**
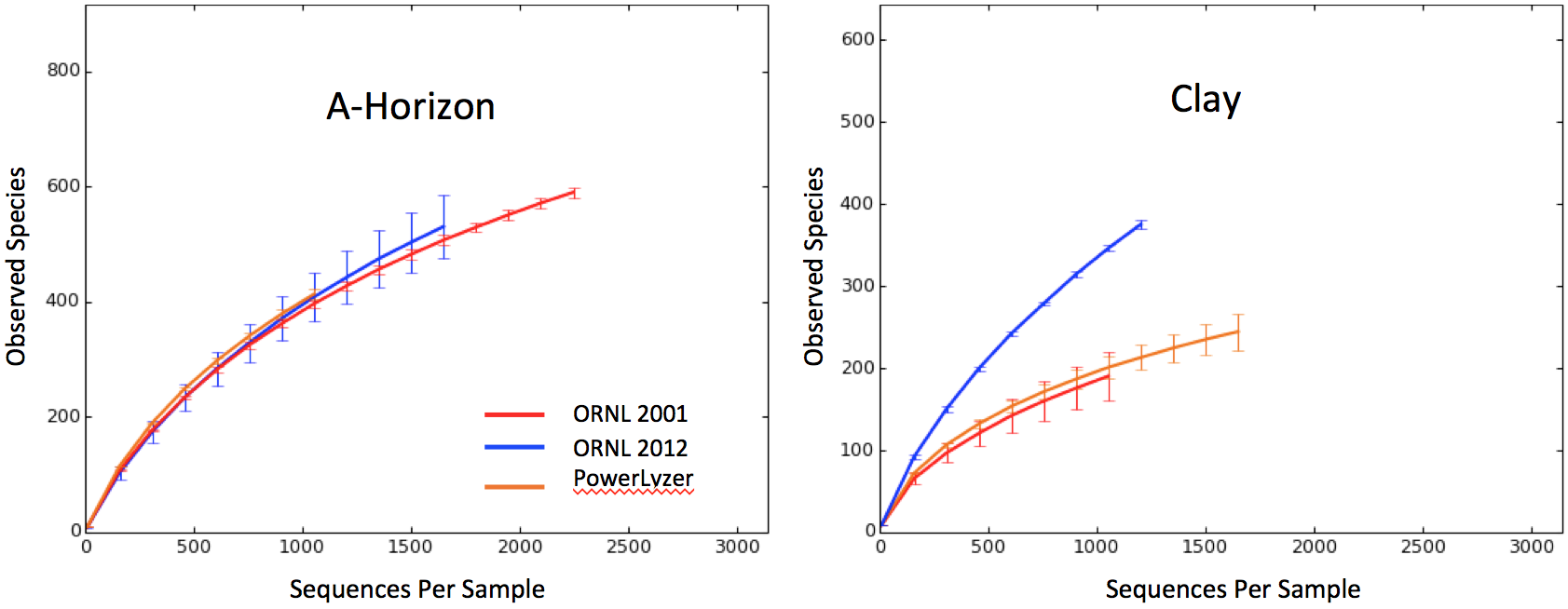
**

**
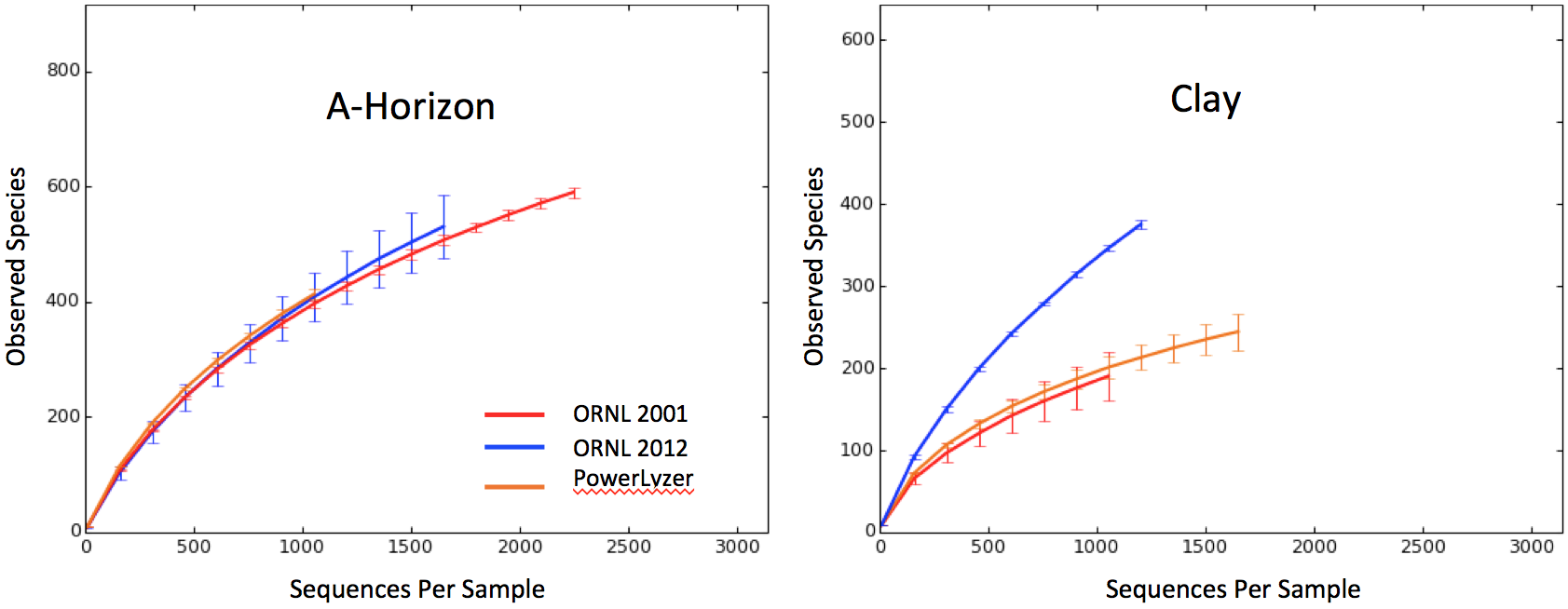
**

**Figure S4. Closed-reference rarefaction curves based on 99% sequence similarity.** Rarefaction curves based on 100 jackknife iterations at each sampling depth for OTUs picked at 99% sequence similarity via closed-reference OTU picking against the Greengenes database [[34](#_ENREF_34),[35](#_ENREF_35),[56](#_ENREF_56)]. If a given sequence did not match within 1% of any sequence within the Greengenes database the sequence was discarded from the analysis.
