# Supplementary material for: Improved Yield of High Molecular Weight DNA Coincides with Increased Microbial Diversity Access from Iron Oxide Cemented Sub-Surface Clay Environments
Source: PLoS One. 2014 Jul 17;9(7):e102826. doi: 10.1371/journal.pone.0102826 (PMC4102596; doi:10.1371/journal.pone.0102826)
Supplement: Figure S5 — Workflow diagrams. (A) The step-wise workflow of the phosphate buffer desportion process to recover DNA that may be bound to particulates. (B) A comparative workflow highlighting the differences between the ORNL2001 and ORNL2012 processes for obtaining high molecular weight DNA. (DOCX) [file pone.0102826.s005.docx]

**B**

**A**

**Figure S5. Workflow diagrams.** (A) The step-wise workflow of the phosphate buffer desportion process to recover DNA that may be bound to particulates. (B) A comparative workflow highlighting the differences between the ORNL2001 and ORNL2012 processes for obtaining high molecular weight DNA.
