# Supplementary material for: Improved Yield of High Molecular Weight DNA Coincides with Increased Microbial Diversity Access from Iron Oxide Cemented Sub-Surface Clay Environments
Source: PLoS One. 2014 Jul 17;9(7):e102826. doi: 10.1371/journal.pone.0102826 (PMC4102596; doi:10.1371/journal.pone.0102826)
Supplement: Table S1 — Basic sample chemistry. (DOCX) [file pone.0102826.s006.docx]

**Table S1. Basic sample chemistry.**

| **Sample** | **Nitrogen %** | **Carbon %** | **Acid Ext. Fe mg/g^a^** | **Fe mg/g^b^** | **Al mg/g^b^** |
| --- | --- | --- | --- | --- | --- |
| **Clay** | 0.078 | 0.370 | 0.08 | 38.66 | 12.30 |
| **A-horizon** | 0.045 | 0.502 | 0.58 | 21.25 | 5.90 |
| **22.3 EFPC** | 0.105 | 1.235 | 2.12 | 7.51 | 4.85 |
| **5.0 EFPC** | 0.106 | 0.737 | 2.16 | 8.16 | 4.13 |
| **Background** | 0.379 | 5.750 | 1.08 | 4.66 | 4.34 |

^a^Acid extractable Fe.

^b^ Extractable Fe or Al following digestion of 0.5 g soil with concentrated HNO_3_ (1 mL) and HCl (0.5 mL).
