# Supplementary material for: Improved Yield of High Molecular Weight DNA Coincides with Increased Microbial Diversity Access from Iron Oxide Cemented Sub-Surface Clay Environments
Source: PLoS One. 2014 Jul 17;9(7):e102826. doi: 10.1371/journal.pone.0102826 (PMC4102596; doi:10.1371/journal.pone.0102826)
Supplement: Table S2 — Extraction method comparison of DNA Yields. (DOCX) [file pone.0102826.s007.docx]

**Table S2. Extraction method comparison of DNA Yields.**

| **Method** | **Clay**  **µg DNA/g** | **A-Horizon**  **µg DNA/g** | **Final Purification Procedure** |
| --- | --- | --- | --- |
| **OU** | 9.63 ± 1.03 | 45.21 ± 6.50 | Gel Purification by Phenol Extract |
| **ORNL2012** | 15.22 ± 2.33 | 189.07 ± 33.69 | Gel Purification/ Promega Wizard Modified Elution |
| **MSU** | 2.31 ± 0.23 | 20.90 ± 3.77 | MoBio PowerSoil™ DNA Cleanup |
| **ORNL2001** | 6.06 ± 3.06 | 10.10 ± 5.46 | Gel Purification/ Promega Wizard |
| **CTABIPA** | 0.00 | 28.86 ± 10.28 | QIAGEN All Prep DNA/RNA |
| **PA-D** | 19.84 ± 1.23 | 182.40 ± 15.26 | Digestion with RNase A |
| **PowerLyzer** | 2.04 ± 0.54 | 13.20 ± 3.48 | PowerLyzer |
